# Genome-Wide Identification of the AP2/ERF Transcription Factor Family and Expression Analysis Under Selenium Stress in *Cardamine hupingshanensis*

**DOI:** 10.3390/biology14121686

**Published:** 2025-11-26

**Authors:** Nanrong Deng, Xixi Zeng, Jialin Liu, Shengcai Chen, Yanke Lu, Zhixin Xiang, Zhi Hou, Qiaoyu Tang, Yifeng Zhou

**Affiliations:** 1 Hubei Key Laboratory of Biological Resources Protection and Utilization, Hubei Minzu University, Enshi 445000, China; 202330426@hbmzu.edu.cn; 2 College of Forestry and Horticulture, Hubei Minzu University, Enshi 445000, China; 3 College of Biological and Food Engineering, Hubei Minzu University, Enshi 445000, China

**Keywords:** *Cardamine hupingshanensis*, AP2/ERF transcription factor, selenium stress, bioinformatics, gene expression

## Abstract

AP2/ERF (APETALA2/ethylene-responsive factor) is one of the largest plant transcription factor families, characterized by 1-2 AP2/ERF domains (≈60–70 amino acids) that regulate plant development and biotic/abiotic stress responses. This study presents the first genome-wide identification and characterization of the AP2/ERF family in the selenium (Se) hyperaccumulator *Cardamine hupingshanensis* via bioinformatics. A total of 230 *AP2/ERF* genes were identified, which were non-randomly distributed across 16 chromosomes. Their encoded proteins varied in length (126–623 aa), molecular mass (13.927–68.112 kDa), and isoelectric point (4.48–10.31). Phylogenetic analysis classified these genes into five conserved subfamilies (AP2, DREB, ERF, RAV, Soloist), consistent with other plant species. Intron distribution differed among subfamilies (42.17% of genes contained introns), and motif 1 was universally conserved. Promoter cis-element analysis revealed enrichment of hormone-, stress-, and growth-related elements, highlighting potential roles in abiotic stress responses (notably, light and abscisic acid signaling). Expression profiling under Se stress (100 μg Se/L and 80,000 μg Se/L) demonstrated tissue-specific, dose-dependent, and temporal dynamic patterns. This inaugural genome-wide investigation of *C. hupingshanensis* AP2/ERFs provides foundational datasets for deciphering regulatory networks governing growth and Se stress response in this Se hyperaccumulator plant.

## Simple Summary

*Cardamine hupingshanensis* is a well-studied selenium (Se) hyperaccumulator plant possessing strong Se tolerance and Se accumulation capabilities. However, the function of its AP2/ERF transcription factor family in mediating Se stress response and Se hyperaccumulation remains unclear. This study conducted the first genome-wide identification and analysis of the AP2/ERF transcription factor family in the Se hyperaccumulator *C. hupingshanensis*. Using bioinformatics methods, a total of 230 *ChAP2/ERF* genes were identified, which were non-randomly distributed across 16 chromosomes. Phylogenetic analysis classified these genes into five subfamilies: AP2, DREB, ERF, RAV, and Soloist. The gene structures and conserved motifs exhibited subfamily-specific characteristics, and the promoter regions were enriched with *cis*-acting elements related to hormones, stress, and growth. Expression analysis under Se stress (100 μg Se/L and 80,000 μg Se/L) showed that the genes of this family displayed tissue-specific, dose-dependent, and temporally dynamic expression patterns. In conclusion, this study can provide a potential basis for subsequent functional analysis of the *ChAP2/ERF* family and also offer fundamental data for deciphering the molecular mechanisms underlying Se stress response in hyperaccumulator plants.

## 1. Introduction

Transcription factors (TFs) serve as core regulators in orchestrating plant growth, development, and stress-responsive signaling cascades. Functioning as “signal integrators”, TFs perceive and transduce external abiotic/biotic stimuli and internal physiological cues to modulate the spatiotemporal expression of target genes, thereby coordinating adaptive physiological and metabolic processes [1]. Among the extensive array of plant TF families, the AP2/ERF (APETALA2/ethylene-responsive factor) superfamily represents one of the largest and most functionally divergent groups, with indispensable roles spanning the entire plant life cycle—from organ initiation and morphogenesis to the mediation of adaptive responses against biotic and abiotic stresses [1,2,3,4]. A defining feature of this superfamily is the presence of a conserved AP2 DNA-binding domain, consisting of approximately 60–70 amino acids. Based on variations in domain architecture and functional specialization, the AP2/ERF superfamily is phylogenetically categorized into five distinct subfamilies: AP2, ERF (ethylene-responsive factor), DREB (dehydration-responsive element-binding protein), RAV, and Soloist [5,6]. The structural heterogeneity of the AP2/ERF superfamily constitutes the molecular foundation for its functional diversification. Specifically, members of the AP2 subfamily harbor two AP2 DNA-binding domains, while the ERF and DREB subfamilies each contain a single AP2 domain [7,8]; the RAV subfamily has evolved a unique bipartite domain structure (AP2-B3); and the Soloist subfamily exhibits a distinct domain organization that distinguishes it from other subfamilies [9]. This structural differentiation directly dictates functional specificity; for example, DREB subfamily proteins are well characterized for their role in mediating responses to dehydration and cold stress through specific binding to dehydration-responsive elements (DREs) in target gene promoters [10,11]. They participate in the regulation of plant growth and development, adaptive responses to abiotic stresses, and the integration of multiple signaling pathways by recognizing and binding to specific *cis*-acting elements [12]. Prior studies of model and crop species, including *Arabidopsis thaliana* (*A. thaliana)*, *Oryza sativa*, and *Brassica napus*, have corroborated that AP2/ERF TFs modulate key physiological processes—such as organ morphogenesis, photosynthetic efficiency, and antioxidant metabolism—by recognizing and binding to distinct *cis*-acting elements in the promoter regions of downstream target genes [4,7,9]. Owing to their structural uniqueness and functional significance in plant growth and stress adaptation, the AP2/ERF superfamily has emerged as a pivotal research focus in plant molecular biology.

Selenium (Se) is an essential trace element for most organisms, yet its effects on plants exhibit a concentration-dependent biphasic pattern, which is further modulated by plant species and Se specialization [13]. At low concentrations, Se can enhance plant stress resistance by reinforcing antioxidant systems and improve photosynthetic efficiency, thereby promoting plant growth. However, when Se concentrations exceed a species-specific threshold, it induces phytotoxicity. This toxicity arises primarily from the structural similarity between Se and sulfur (S), leading to competitive inhibition of S transporters and metabolic enzymes, which, in turn, triggers the overproduction of reactive oxygen species (ROS) and subsequent growth inhibition [14,15]. Most plant species display evident Se toxicity symptoms under high Se exposure. In contrast, a specialized group of plants termed “Se hyperaccumulators” has evolved extraordinary Se tolerance and the capacity to accumulate Se to levels 10–100 times higher than non-accumulating counterparts, making them ideal models for dissecting the molecular mechanisms underlying extreme Se adaptation [16].

*Cardamine hupingshanensis*, a Brassicaceae species endemic to selenium-rich aquatic habitats in Hubei Province, China, is a typical Se hyperaccumulator with exceptional Se tolerance and accumulation capabilities [17,18,19]. Unlike non-hyperaccumulating plants, which suffer from severe growth inhibition under high-Se conditions, *C. hupingshanensis* has evolved a specialized regulatory network that prioritizes Se uptake, translocation, and detoxification while maintaining normal growth and development [14,19,20]. Existing studies on the Se adaptation mechanisms of *C. hupingshanensis* have provided preliminary insights: Zhou et al. employed comparative transcriptomics to identify differentially expressed genes (DEGs) involved in Se transport and antioxidant defense [19]; Cui et al. elucidated the patterns of Se translocation and specialization transformation across different tissues [18]; and Zeng et al. characterized key enzymes in the selenographer cycle [20], suggesting a critical role of metabolic regulation in Se detoxification. Additionally, the structural homology between Se and S implies that *C. hupingshanensis* may have modified S metabolism-related pathways to mitigate Se-S competition [21]. Despite these advances, the upstream transcriptional regulatory networks that govern these Se-responsive processes remain largely uncharacterized. Although previous studies have preliminarily uncovered the adaptive mechanisms of *C. hupingshanensis* in selenium (Se) uptake, translocation, and metabolism, the role of the core AP2/ERF transcription factor family in Se stress response and hyperaccumulation remains uncharacterized.

This study represents the first genome-wide investigation and comprehensive characterization of the AP2/ERF transcription factor family in *C. hupingshanensis*. Focusing on this species with exceptional selenium (Se) tolerance and accumulation capabilities, we systematically elucidated the physicochemical properties and functional bases of 230 identified *ChAP2/ERF* genes, and uncovered species-specific evolutionary traits, by integrating phylogenetic analysis, conserved motif characterization, gene structure annotation, chromosomal mapping, and duplication event detection. This research not only facilitates the identification of core regulatory genes governing the growth and development of *C. hupingshanensis* but also provides a novel perspective revealing the adaptive strategies of plants in response to Se stress.

## 2. Methods

### 2.1. Genome-Wide Identification of ChAP2/ERF Genes

The gene annotation GTF file, nucleotide sequence FASTA file, and protein sequence FASTA file of *C. hupingshanensis* were downloaded from the Genome Warehouse BIG Data Centre (https://ngdc.cncb.ac.cn/gwh/, accessed on 5 May 2024, PRJCA005533). The protein sequences of *A. thaliana* were obtained from The Arabidopsis Information Resource (https://www.arabidopsis.org/, accessed on 5 May 2024), which were used as a query sequence for extracting the homologous protein sequence using the Blast Zone (BlastType:blastp, Outfmt: Table) of TBtools software (v2.154) [22]. The obtained protein sequences were further verified using NCBI BLAST 2.17.0 (https://blast.ncbi.nlm.nih.gov/Blast.cgi, accessed on 5 May 2024). The conserved domain of proteins was further analyzed by CD-search (https://www.ncbi.nlm.nih.gov/Structure/bwrpsb/bwrpsb.cgi, accessed on 7 May 2024). The physical and chemical properties of molecular weight (MW), isoelectric point (pI), grand average of hydropathicity (GRAVY), and instability index were predicted and analyzed by the online tool ExPASy (https://web.expasy.org/protparam/, accessed on 20 May 2024) [23]. The subcellular localization was predicted by WoLF PSORT (https://wolfpsort.hgc.jp/, accessed on 20 May 2024).

### 2.2. Chromosomal Distribution and Phylogenetic Analysis of ChAP2/ERF Genes

The chromosomal location information was obtained from the gene annotation GTF file of *C. hupingshanensis* for visualization by “Gene Location Visualize from GTF/GFF” in the TBtools software. The protein sequences of *A. thaliana* were downloaded from the NCBI (https://www.ncbi.nlm.nih.gov/, accessed on 20 May 2024) for multiple sequence alignment by Clustal W. A maximum likelihood (ML) tree with *C. hupingshanensis* and *A. thaliana* was constructed with all of the protein sequences using MEGA 11, bootstrap = 1000 repetitions. The resulting multi-species phylogenetic trees were visualized and annotated using Evolview: Tree View (https://www.evolgenius.info/evolview/#/treeview, accessed on 25 May 2024).

### 2.3. Structure and Functional Characteristics Analysis of ChAP2/ERF Genes

The protein sequences were submitted to the MEME website (http://meme-suite.org/tools/meme, accessed on 1 June 2024) to perform a conserved motif scan with the MEME motif set to 20. The conserved domain information was obtained in the CD-search of the NCBI’s conserved domain database (https://www.ncbi.nlm.nih.gov/Structure/cdd/wrpsb.cgi, accessed on 1 June 2024) by submitting the protein sequences. The intron–exon gene structure information of genes was extracted from the GFF files of the *C. hupingshanensis* and *A. thaliana* genomes for further visualization by “Gene Structure View (advanced)” of TBtools [22]. The protein sequences of *C. hupingshanensis* and *A. thaliana* were aligned by ClustalW (https://www.genome.jp/tools-bin/clustalw, accessed on 9 June 2024). The result was further processed by ESPript 3.0 (https://espript.ibcp.fr/ESPript/cgi-bin/ESPript.cgi accessed on 9 June 2024) to output the image [24].

### 2.4. Analysis of Cis-Acting Elements in the ChAP2/ERF Family

To identify *cis*-regulatory elements in the promoters of *ChAP2/ERF* genes, 2000 bp sequences upstream of the ATG start codon were extracted using TBtools. These sequences were submitted to the PlantCARE database (http://bioinformatics.psb.ugent.be/webtools/plantcare/html/, accessed on 15 June 2024) for the prediction of *cis*-regulatory elements. The results were compiled and visualized using TBtools.

### 2.5. Collinearity Relationship and Identification of Gene Duplication Events

Intra-genomic collinearity analysis of the *C. hupingshanensis* genome (GFF3 files) was conducted using MCScanX integrated into TBtools (default parameters), identifying tandem and segmental duplications among *ChAP2/ERF* genes. Cross-species synteny between *C. hupingshanensis* and *A. thaliana* was analyzed by aligning their genomes and GFF3 files via MCScanX, generating control (ctl), GFF, and collinearity files. Mitochondria and chloroplast sequences were excluded, and results were visualized using TBtools [22]. Sequences (CDS) of duplicated gene pairs were aligned to evaluate evolutionary selection pressure, and Ka/Ks ratios were calculated using TBtools’ Ka/Ks module with the Nei–Gojobori method. Gene pairs were classified under purifying (Ka/Ks < 1) or positive selection (Ka/Ks > 1).

### 2.6. Plant Material and Sample Preparation

The seeds of *C. hupingshanensis* were collected from the 5th floor of the Key Laboratory of Hubei University for Nationalities, Enshi, Hubei Province. The *C. hupingshanensis* seeds were planted in a chamber at 22 ± 1 °C with a 16/8 h light/dark photoperiod, and the irradiance was 1500 μmol·m^−2^·s^−1^. Forty-five uniform seedlings, approximately 10 cm in height and 4 months old, were selected as experimental materials. Root systems of the seedlings were carefully rinsed with deionized water to remove residual impurities and then transferred to Hoagland’s nutrient solution for acclimatization culture for two days. Selenium-containing solutions were prepared using sodium selenite (Na_2_SeO_3_) at final concentrations of 100 μg Se·L^−1^ and 80,000 μg Se·L^−1^. Seedlings treated with Hoagland’s nutrient solution without selenium (0 μg Se·L^−1^) served as the control group. After acclimatization, seedlings were exposed to the aforementioned selenium solutions. Leaf and root samples were collected at 0, 3, 6, 12, and 24 h post-treatment. When collecting samples, root systems were first rinsed with deionized water. Excess water was blotted dry with filter paper, and then leaves and root systems were separated using cleaned scissors. The samples were wrapped in tinfoil, immediately snap-frozen in liquid nitrogen, and stored at −80 °C until RNA extraction. RNA isolation for all samples was performed within 72 h of collection. Three biological replicates were set for each treatment group and the control group to ensure the reliability and reproducibility of the experimental results.

### 2.7. Gene Expression Analysis

The TransZolTM Up Plus RNA Kit (TransGen Biotech, Beijing, China) was used to extract the total RNA of roots and leaves, and the RNA concentration and quality were detected by a NanoDrop 2000 (Thermo Fisher Scientific, Waltham, MA, USA). RNA integrity and genomic DNA contamination were analyzed by 1% agarose gel electrophoresis. RNase-free DNase removed residual genomic DNA in RNA samples. Real-time PCR was carried out on the ABI StepOne Plus (Thermo Fisher Scientific, Waltham, MA, USA). Detection of the target gene expression levels in the samples was facilitated by the Hieff qPCR SYBR Green Mix reagent kit (Yeasen Biotechnology, Shanghai, China). The ChActin gene was used as an internal reference for normalization, and relative gene expression was calculated using the 2^−∆∆CT^ method [25]. The results were analyzed, and a graphical representation was created using GraphPad Prism (Version 9.0, GraphPad Software, Inc.), while the significance was meticulously evaluated through the LSD test of single-factor ANOVA (*p* < 0.05) [26]. All analyses were conducted in triplicate to ensure reproducibility and accuracy. The primers used for the qRT-PCR analysis are listed in Table S2.

### 2.8. GO/KEGG Enrichment Analysis of ChAP2/ERF Targets

Target genes with the ERF protein binding site elements, DRE/CRT (G/ACCGAC) and GCC-box (AGCCGCC), were investigated using TBtools. Gene Ontology (GO) analysis and Kyoto Encyclopedia of Genes and Genomes (KEGG) Enrichment analyses of target genes were performed using eggNOG-mapper v2 (http://eggnog-mapper.embl.de/, accessed on 9 December 2024) and visualized using Tbtools.

### 2.9. Statistical Analysis

Statistical analyses were conducted using SPSS (v27.0.1; IBM Corp.) with one-way ANOVA followed by LSD post hoc tests (significance threshold: *p* < 0.05). All data represent mean values ± standard deviation (*n* = 3 biological replicates). Graphical preparation was performed using GraphPad Prism software and Adobe Illustrator 2021 software [27].

## 3. Results

### 3.1. Identification and Characteristic Analysis of ChAP2/ERF in C. hupingshanensis

A total of 230 genes were identified in *C. hupingshanensis* (the Genome Warehouse BIG Data Centre accession number PRJCA005533) by comparison with the genome sequences of *A. thaliana*, including 32 *ChAP2* genes, 71 *ChDREB* genes, 114 *ChERF* genes, 11 *ChRAV* genes, and 2 *ChSoloist* genes (Table 1). The sequences and coding regions of each protein are listed in Table S1. The protein length in *C. hupingshanensis* ranged from 126 aa (*ChERF011-2*) to 623 aa (*ChAP2-1*), with an average of 273 amino acids. Consistent with the number of amino acids, the shortest coding sequence contained 381 bp (*ChERF011-2*), and the longest only 1872 bp (*ChAP2-1*). The molecular weight (MW) ranged from 13.927 (*ChERF011-2*) to 68.112 kDa (*ChAP2-1*), and the isoelectric point (PI) ranged from 4.48 (*ChERF016-2*) to 10.31 (*ChERF072-2*). It is worth noting that all proteins were hydrophilic except for *ChERF087-2.* About 80.9% (186 genes) of the *ChAP2/ERF* genes are mainly located in the nucleus. A small number of genes, about 12.2% (28 genes), are located in the chloroplast, about 2.6% (6 genes) in the cytosol, and about 2.2% (5 genes) in the mitochondria. Significantly, *ChERF007* (two genes) is located in the peroxisome, *ChAP2-26* is located in the extracellular space, *ChERF108-2* is located in the plasma membrane, and *ChRAV6-2* is situated in the cytoskeleton. From the above information regarding the different characteristics in *AP2/ERF* genes, we could speculate that *AP2/ERF* genes have versatile roles in *C. hupingshanensis.*

### 3.2. Phylogenetic Analysis of ChAP2/ERF in C. hupingshanensis

**Figure 1:**
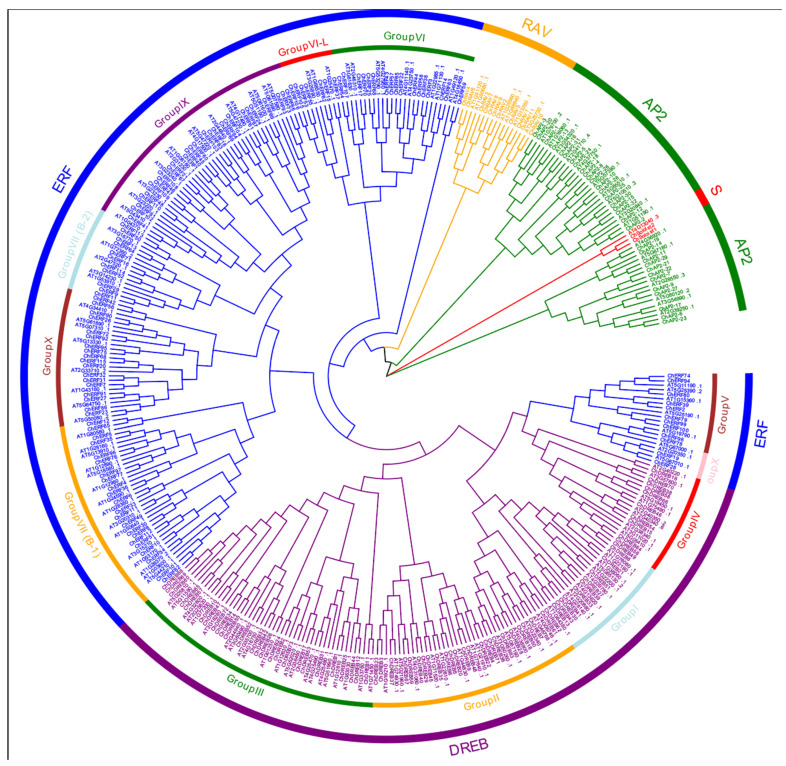
Phylogenetic tree of the *AP2/ERF* family in *A. thaliana* and *C. hupingshanensis* (Ch). S is *Soloist*. Different colors represent different subfamilies.

To analyze the evolutionary relationship of the *AP2/ERF* family in *C. hupingshanensis*, the phylogenetic trees of 166 *AP2/ERF* genes in *A. thaliana* and 230 in *C. hupingshanensis* proteins were constructed by maximum likelihood (ML) based on multiple sequence alignments (Figure 1). Referring to the classification of *AP2/ERF* genes in *A. thaliana*, all genes from *C. hupingshanensis* were divided into five primary subfamilies: *AP2*, *DREB*, *ERF*, *RAV*, and *Soloist*. The genes of the *ERF* subfamily were further classified into subgroups V, VI, VI-L, VII, VIII, IX, and X, and the subgroups of the *DREB* subfamily were I, II, III, and IV. The phylogenetic distribution showed that the *ChERF* subfamily contained most genes, about 49.6%, followed by *ChDREB* with about 30.9%, the *ChAP2* subfamily with about 13.9%, and the *RAV* subfamily and *Soloist* subfamily with about 5.7%, which was made up of the fewest genes. It is worth noting that Group I of the *DREB* subfamily was distributed in two distinct clades and Group Xb-L of the *ERF* subfamily had relative independence. These results indicate that the AP2/ERF family in *C. hupingshanensis* maintains a certain degree of evolutionary conservation with *A. thaliana* while simultaneously forming species-specific subgroup differentiation. This phenomenon may be associated with the specific functional divergence of these subgroups in responding to environmental stresses or regulating the unique growth and development processes of this selenium hyperaccumulator species.

### 3.3. Chromosomal Distribution and Gene Duplication and Synteny Analysis of ChAP2/ERF Family

**Figure 2:**
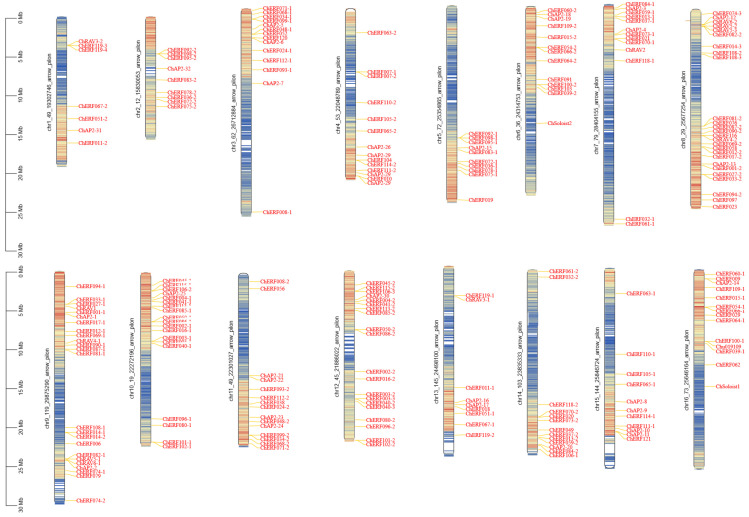
Chromosomal distribution of *AP2/ERF* genes in *C. hupingshanensis*. The chromosome numbers are shown on the left side of each strip. Chromosome colors represent gene abundance.

Chromosomal distribution analysis indicated that the 230 *ChAP2/ERF* genes were unevenly distributed on 16 chromosomes (Figure 2). The largest number of *ChAP2/ERF* genes was found on chromosomes 8 and 9 (25 and 24 genes, respectively), while chromosome 1 owned the smallest number of *ChAP2/ERF* genes (7 genes). Only *ChERF* and *ChAP2* members were found on chromosome 5. Eleven *ChRAV* subfamily members were distributed on chromosomes 1, 7, 8, 9, and 13, and two *ChSoloist* subfamily members were distributed on chromosomes 6 and 16. Chromosomes 6, 7, and 16 were all located on 13 genes, and chromosomes 3 and 4 were all located on 14 genes.

**Figure 3:**
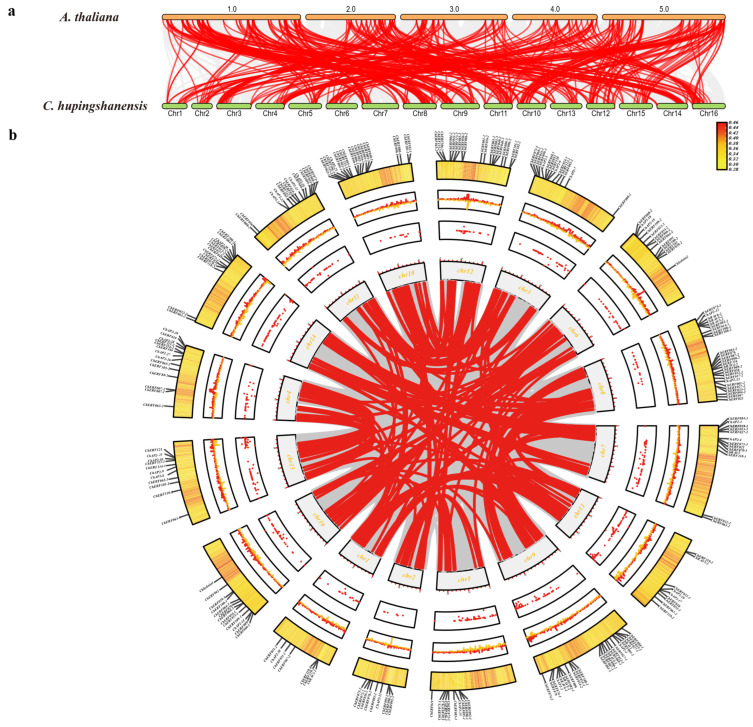
Synteny and Evolutionary Analysis of the *ChAP2/ERF* Gene Family. (**a**) Synteny analysis of *ChAP2/ERF* genes between *C. hupingshanensis* and *A. thaliana*. Red lines represent collinear *ChAP2/ERF* gene pairs, while gray lines denote all syntenic blocks within the genomes. (**b**) Intra-genomic collinearity map of *ChAP2/ERF.* In the Circos plot, progressing from inner to outer rings, the gray lines in the background represent collinear regions within the *C. hupingshanensis* genome. In contrast, the red lines highlight collinear gene pairs specific to *ChAP2/ERF*. The figure also includes a dot plot showing N-ratio distribution, a line plot of GC skew, a heatmap of gene density distribution, and a line plot depicting GC content variation. The labels indicate the *ChAP2/ERF* gene family.

Homologous gene identification revealed that there were 72 pairs of *AP2/ERF* homologous genes between *C. hupingshanensis* and *A. thaliana*. Subsequently, we implemented a collinearity analysis of the *AP2/ERF* gene families of *C. hupingshanensis* and *A. thaliana* (Figure 3). The results showed that there were multiple homologous genes between *C. hupingshanensis* and *A. thaliana*, which were distributed on 16 chromosomes. There were multiple tandem repeat regions on each chromosome. This may imply that, during the evolutionary process, the related genes underwent multiple duplications, which is likely an evolutionary strategy adopted by plants to adapt to environmental changes. In *C. hupingshanensis,* we found that all the 230 genes had undergone multiple segmental duplication events, and they were distributed on 16 chromosomes (Figure 3). Different numbers of genes that had undergone tandem duplication belonged to different chromosomes. Chromosome 8 had the largest number of genes, with 25 genes undergoing tandem duplication, while chromosome 1 had the fewest, with only 7. Genes from the same subfamily were not located on the same chromosome, but gene duplication phenomena existed. In summary, this study analyzed the chromosomal distribution of 230 *ChAP2/ERF* genes in *C. hupingshanensis*, revealing an uneven pattern with the highest gene counts on chromosomes 8 and 9, the lowest on chromosome 1, and subfamily-specific localization characteristics. Additionally, 72 pairs of *AP2/ERF* homologous genes were identified between *C. hupingshanensis* and *A. thaliana*, and widespread segmental and tandem duplication events were detected (chromosome 8 harbored the most tandemly duplicated genes, while chromosome 1 had the fewest). These findings provide valuable insights into the evolutionary characteristics of the *AP2/ERF* gene family in *C. hupingshanensis*.

### 3.4. Structure and Functional Characteristics Analysis of ChAP2/ERF Family

**Figure 4:**
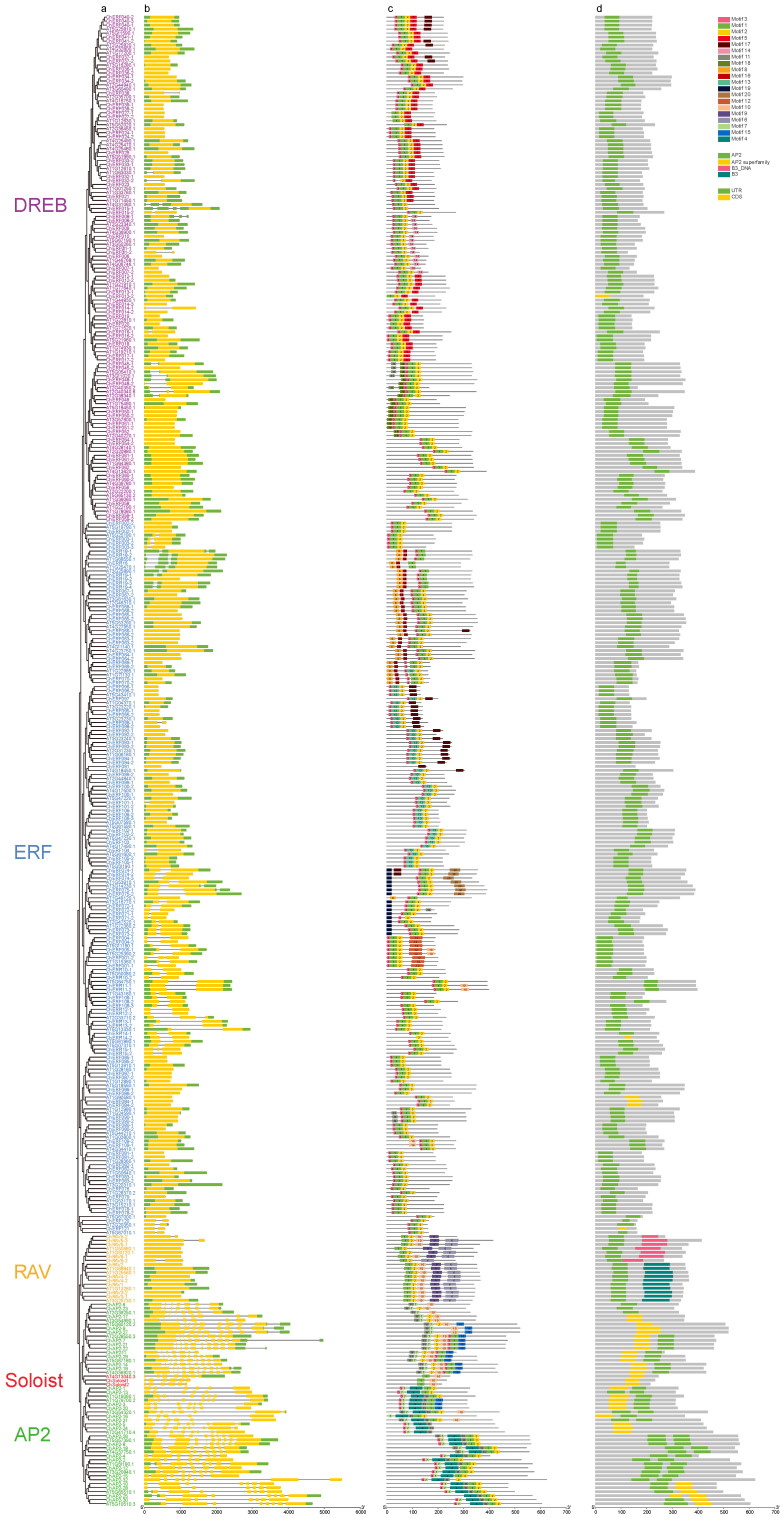
Phylogenetic trees, motifs, domains, and gene structures of *ChAP2*: (**a**) the phylogenetic tree; (**b**,**c**) exon–intron structures, where exons are indicated by yellow boxes and introns are indicated by lines; (**d**) conserved motifs and domains of the proteins, where different colors represent different motifs or domains.

To better understand the evolutionary relationships and structural components of the *ChAP2/ERF* superfamily, the exon–intron gene structures based on genome sequences and conserveed motifs based on protein sequences were analyzed (Figure 4). Structural analysis suggested that the number of introns among different subfamily genes varied markedly. Of the *AP2/ERF* genes in *C. hupingshanensis,* 42.17% had at least one intron. Nearly all genes of the *ChAP2* subfamily (except for *ChAP2-32*) had an intron number ranging from 4 to 10. In the *ChDREB* subfamily, 11 genes had an intron number ranging from 1 to 3; 48 genes of the *ChERF* subfamily had 1 or 2 introns; 4 genes of the *ChRAV* subfamily had 1 intron; and all genes of the *ChSoloist* subfamily had 5 introns. Interestingly, not all *ChAP2/ERF* genes had untranslated regions (UTR). Furthermore, the genes clustered into the same branch on the phylogenetic tree had similar lengths of coding regions and exon–intron structures. The results indicated that the functions of the *ChAP2/ERF* family were relatively conserved during evolution, but functional differentiation also existed.

Different subfamilies had significant differences in the types and distribution of conserved motifs, while genes of the same subclass had similar conserved motifs. For example, motifs 1 to 25 were present in all *ChAP2/ERF* protein sequences except for the *ChRAV* (motifs 1 to 23) and *ChSoloist* (motifs 1 to 4) subfamilies. Motif 1 was present in all *ChAP2/ERF* protein sequences. The type and distribution of motifs of genes in the same branch were relatively similar, while the characteristics of motifs in different branches were quite different, implying that the evolution of *ChAP2/ERF* genes was both conservative and functionally differentiated.

**Figure 5:**
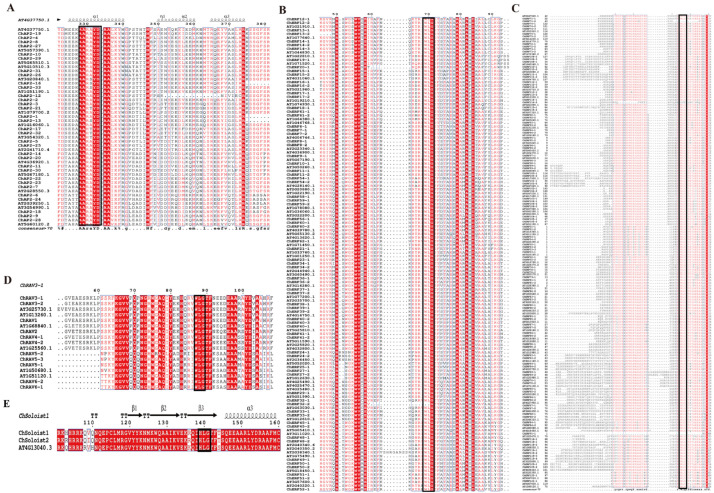
Multiple alignments of partial sequences of the *C. hupingshanensis* AP2/ERF proteins. Secondary structure elements are defined according to ESPript3.0 [24], with helixes representing alpha helices and arrows representing beta strands. The black box marks the conserved catalytic domain. (**A**–**E**).

The conserved domains of *ChAP2/ERF* proteins were predicted. The results showed that the 32 genes of the *ChAP2* subfamily all contained two AP2 domains (AP2/AP2 superfamily, CL00033), and the 11 genes of the *ChRAV* subfamily contained one AP2 domain and one B3 domain. All the genes of the *ChDREB* and *ChERF* subfamilies contained only one AP2 domain. The AP2/ERF domain is conserved among the *ChAP2/ERF* family of transcription factors. AP2 subfamily proteins of this subfamily contain two AP2/ERF domains in their sequences but lack the WLG motif (Figure 5A). Previous studies have reported that, in AP2/ERF transcription factors of other plant species, the WLG motif possesses dual functions; it not only stabilizes the three-dimensional conformation of the AP2 domain to ensure the efficiency of DNA binding, but also mediates the transactivation activity of these transcription factors through interactions with downstream co-regulatory proteins [28]. The *ERF* and *DREB* subfamilies possess a single AP2/ERF domain with a well-conserved WLG motif (Figure 5B,C). In the case of the *RAV* subfamily, apart from the AP2/ERF domain and conserved WLG motif, an additional conserved B3 domain can be observed at the C-terminus region (Figure 5D). The *Soloist* subfamily also possesses the AP2/ERF domain without the WLG motif (Figure 5E). In summary, we analyzed the exon–intron structures, conserved motifs, and conserved domains of the *ChAP2/ERF* superfamily in *C. hupingshanensis*, revealing subfamily-specific differences in intron number, motif types/distribution, and domain composition, as well as conservation in same-branch genes, which indicates both evolutionary conservation and functional differentiation of the *ChAP2/ERF* superfamily.

### 3.5. Putative Promoter Regions Analysis of ChAP2/ERF Family

**Figure 6:**
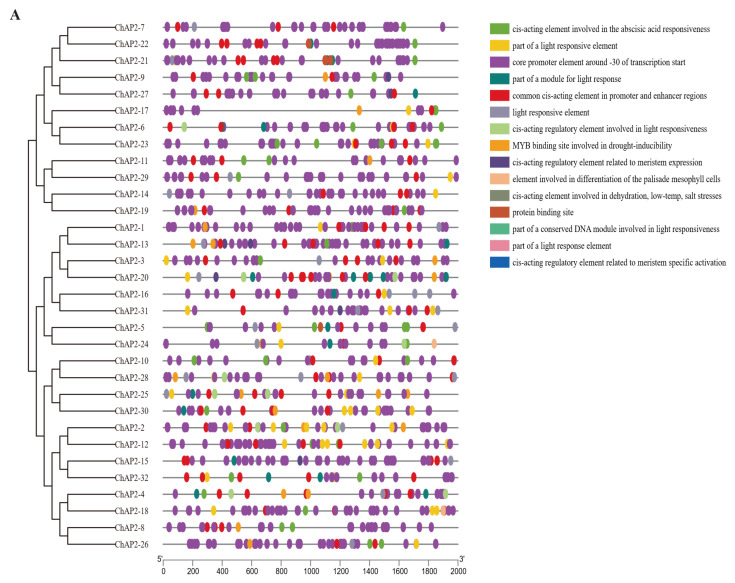

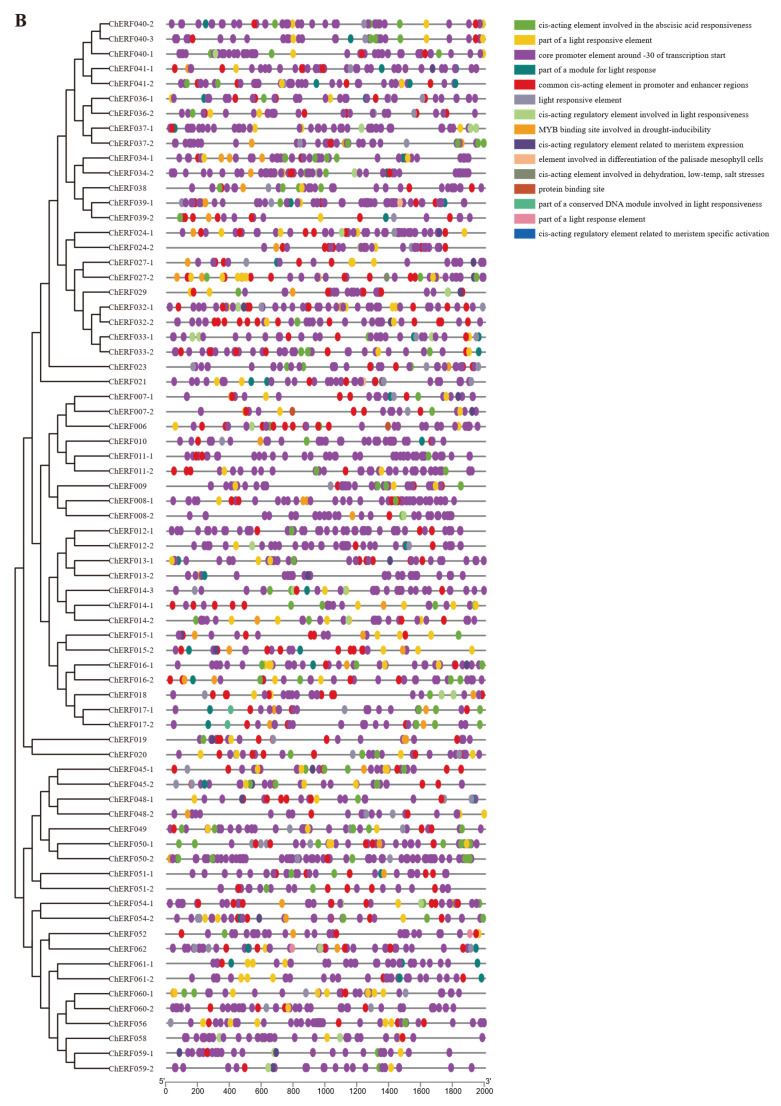

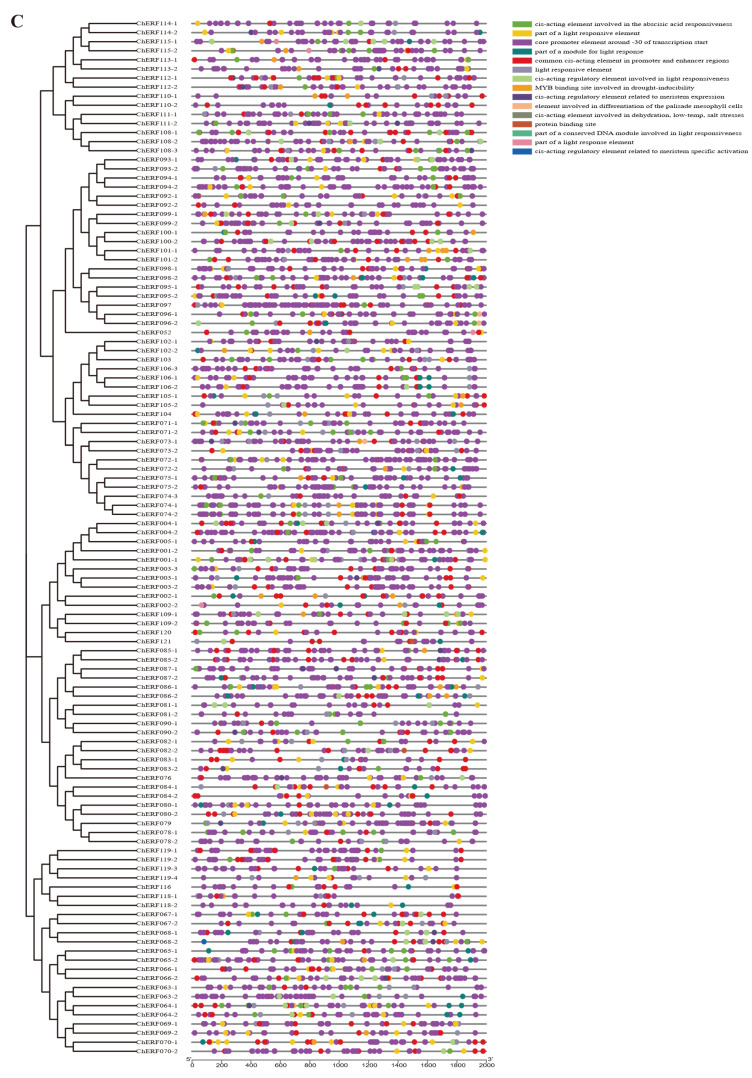

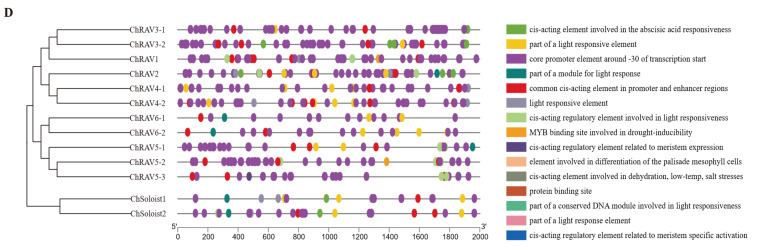
*cis*-acting elements and phylogenetic trees in the promoter region of *ChAP2/ERF* genes. The 2000 bp promoter region upstream of the gene was analyzed. Different colored circles represent different *cis*-acting elements. ((**A**): the *ChAP2* subfamily, (**B**): the *ChDREB* subfamily, (**C**): the *ChERF* subfamily, (**D**): the *ChRAV* and *ChSoloist* subfamilies).

PlantCARE was used to analyze the sequence located upstream of the *ChAP2/ERF* gene, about 2000 bp, and the results showed that the promoter regions of *ChAP2/ERF* family genes contained a variety of *cis*-acting elements (Figure 6). It can be roughly divided into four categories: hormone response, stress response, growth and development-related elements, and other unclassified elements. Among them, the hormone response elements included only abscisic acid elements, the stress response elements included dehydration, low temperature, salt stress, drought defense, and other related elements, and the growth and development-related elements included meristem, palisade mesophyll cells, and other elements. Most of the other unclassified elements included core promoter elements (around −30 of transcription start, 9053) and common *cis*-acting elements in promoter and enhancer regions (971). Therefore, it is speculated that the *ChAP2/ERF* gene plays an important role in the process of abiotic stress, especially in light response and abscisic acid response. In summary, through the analysis of the approximately 2000 bp sequence upstream of the *ChAP2/ERF* genes in *Buddleja hupingshanensis* using PlantCARE, a variety of *cis*-acting elements were identified, including hormone response elements, stress response elements, growth and development-related elements, and other elements. This indicates that the *ChAP2/ERF* genes play an important role in abiotic stress, especially in light response and abscisic acid response.

### 3.6. Expressions Analysis of ChAP2/ERF Family in Different Tissues Under Se Stress

**Figure 7:**
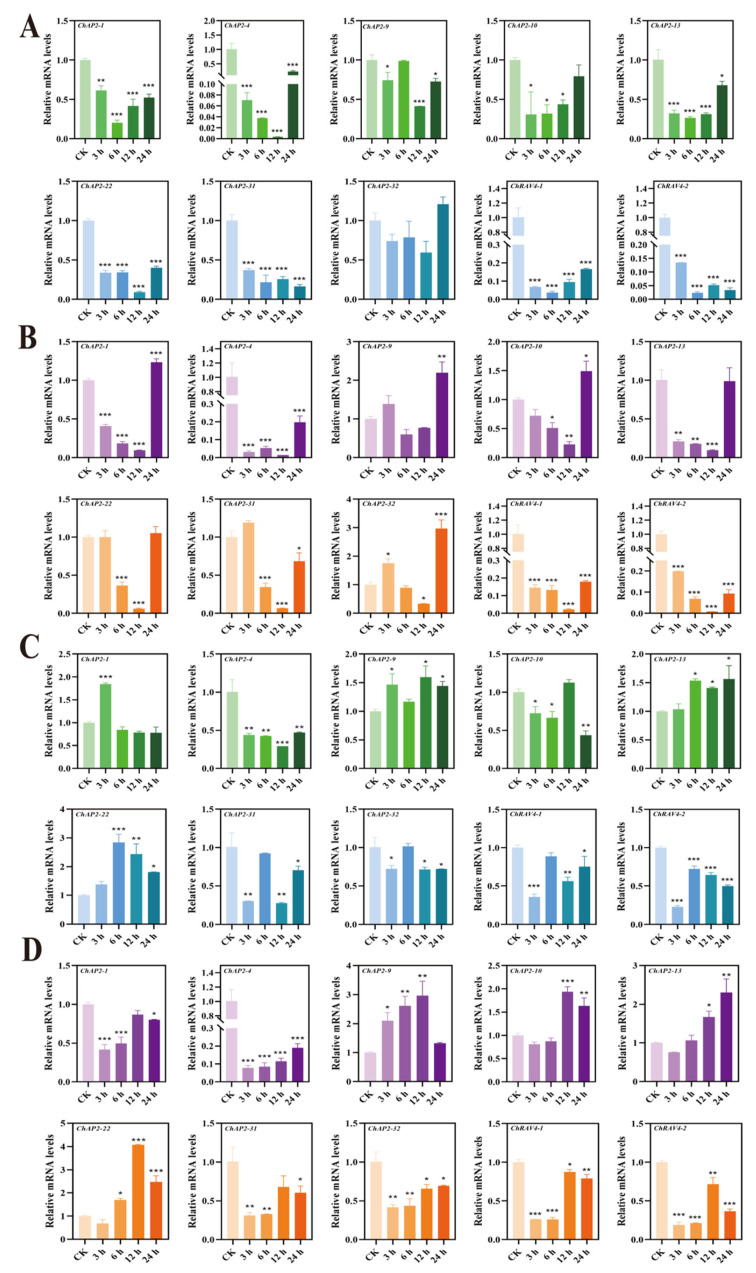
Expression of *ChAP2* and *ChRAV* family genes under different concentrations of selenium stress and in different tissues. (**A**) Expression of *ChAP2* and *ChRAV* family genes in leaves under low-concentration selenium stress (100 μgSe/L). (**B**) Expression of *ChAP2* and *ChRAV* family genes in leaves under high selenium stress (80,000 μgSe/L). (**C**) Expression of *ChAP2* and *ChRAV* family genes in roots under low-concentration selenium stress (100 μgSe/L). (**D**) Expression of *ChAP2* and *ChRAV* family genes in roots under high selenium stress (80,000 μgSe/L). The abscissa represents genes and the ordinate represents the relative expression levels at different time points (0 (control group), 3, 6, 12, and 24 h) under different treatments (0 µgSe/L, 100 µgSe/L, and 80,000 µgSe/L). Each data point represents the mean ± standard deviation (SD) (*n* = 3). Error bars represent the standard deviation. “*” represents a *p*-value less than or equal to 0.05; “**” represents a *p*-value less than or equal to 0.01; “***” represents a *p*-value less than or equal to 0.001.

**Figure 8:**
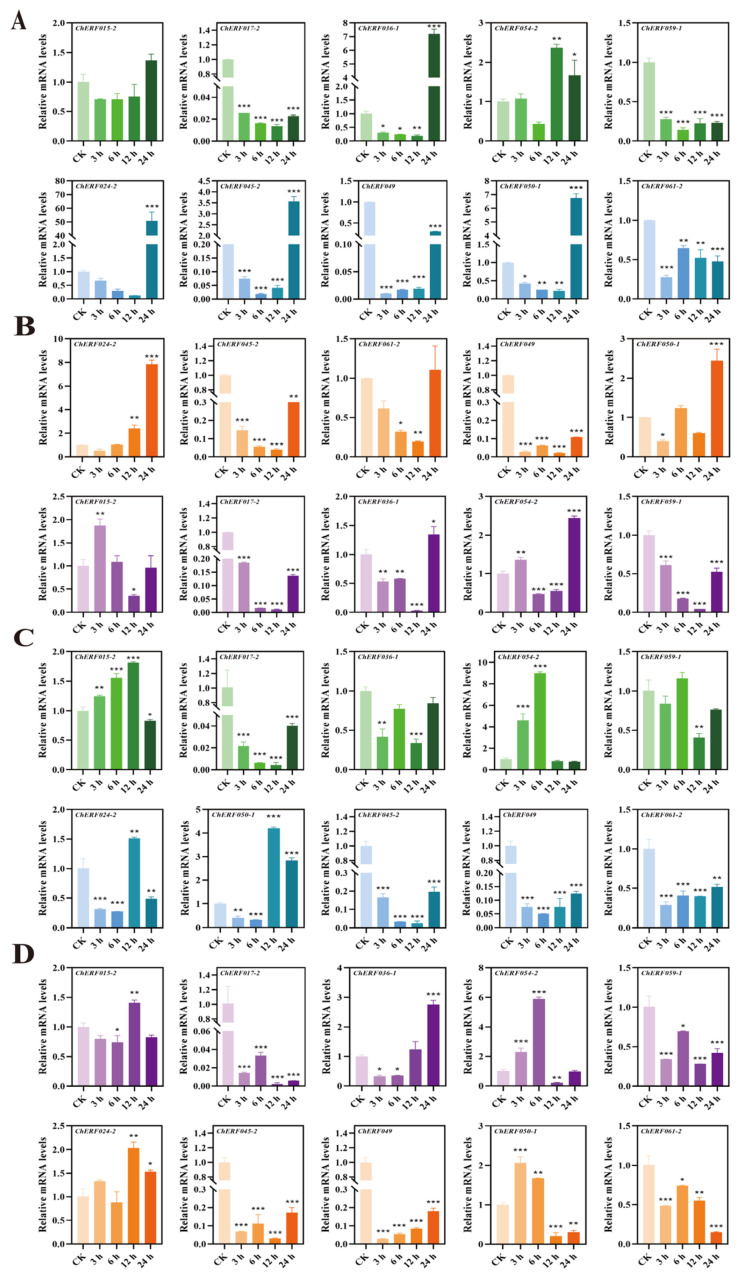
Expression of *ChDREB* family genes under different concentrations of selenium stress and in different tissues. (**A**) Expression of *ChDREB* family genes in leaves under low-concentration selenium stress (100 μgSe/L). (**B**) Expression of *ChDREB* family genes in leaves under high selenium stress (80,000 μgSe/L). (**C**) Expression of *ChDREB* family genes in roots under low-concentration selenium stress (100 μgSe/L). (**D**) Expression of *ChDREB* family genes in roots under high selenium stress (80,000 μgSe/L). The abscissa represents genes and the ordinate represents the relative expression levels at different time points (0 (control group), 3, 6, 12, and 24 h) under different treatments (0 µgSe/L, 100 µgSe/L, and 80,000 µgSe/L). Each data point represents the mean ± standard deviation (SD) (*n* = 3). Error bars represent the standard deviation. “*” represents a *p*-value less than or equal to 0.05; “**” represents a *p*-value less than or equal to 0.01; “***” represents a *p*-value less than or equal to 0.001.

**Figure 9:**
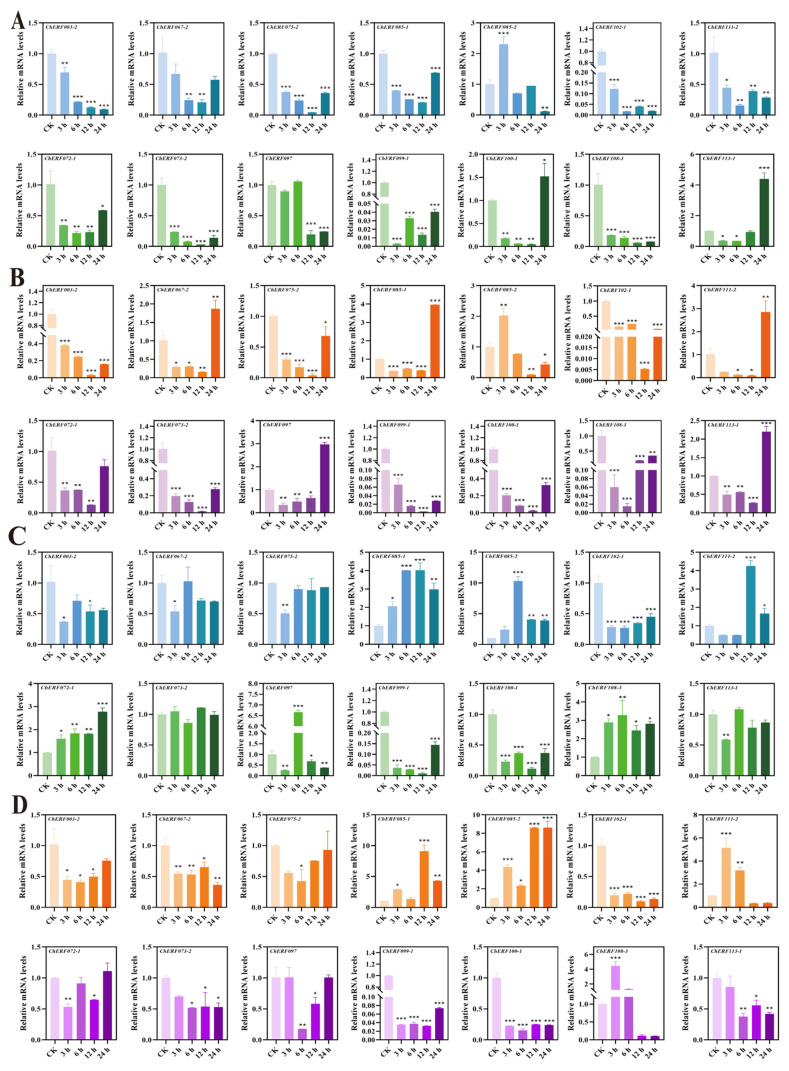
Expression of *ChERF* family genes under different concentrations of selenium stress and in different tissues. (**A**) Expression of *ChERF* family genes in leaves under low-concentration selenium stress (100 μgSe/L). (**B**) Expression of *ChERF* family genes in leaves under high selenium stress (80,000 μgSe/L). (**C**) Expression of *ChERF* family genes in roots under low-concentration selenium stress (100 μgSe/L). (**D**) Expression of *ChERF* family genes in roots under high selenium stress (80,000 μgSe/L). The abscissa represents genes and the ordinate represents the relative expression at different time points (0 (control group), 3, 6, 12, and 24 h) under different treatments (0 µgSe/L,6,6, Se6,16, d 80,000 µgSe/L). Each data point represents the mean ± standard deviation (SD) (*n =* 3). Error bars represent the standard deviation. “*” represents a *p*-value less than or equal to 0.05; “**” represents a *p*-value less than or equal to 0.01; “***” represents a *p*-value less than or equal to 0.001.

qRT–PCR technology was used to detect the expression of some *AP2/ERF* genes in the leaves and roots of *C. hupingshanensis*. As the seedlings of *C. hupingshanensis* treated with 100 μgSe/L selenite arrived at 24 h, *ChAP2-32* was upregulated 1.2-fold in leaves, and other genes were downregulated (Figure 7A). *ChERF024-2* was significantly upregulated 50.8-fold in leaves, and the expression levels of *ChERF036-1*, *ChERF045-2,* and *ChERF050-1* were shown to be highly upregulated by more than 3.5-fold at 24 h (Figure 8A). *ChERF085-2* was upregulated 2.3-fold at 3 h and *ChERF113-1* was upregulated 4.4-fold at 24 h in leaves (Figure 9A). For roots, *ChAP2-32* was upregulated approximately 2.8-fold at 6 h, and *ChAP2-1* was upregulated approximately 1.8-fold at 3 h (Figure 7C). *ChERF050-1* was upregulated 4.2-fold at 12 h and *ChERF054-2* was upregulated 9-fold at 12 h (Figure 8C). *ChERF085-2* was upregulated 10.3-fold and *ChERF097* was upregulated 6.7-fold at 6 h (Figure 9C).

When the seedlings of *C. hupingshanensis* were treated with 80,000 μgSe/L selenite, *ChAP2-9* genes were upregulated 2.2-fold and *ChAP2-32* was upregulated 3-fold at 24 h in leaves (Figure 7B). *ChERF024-2* was upregulated 7.8-fold and *ChERF050-1* and *ChERF054-2* were upregulated 2.5-fold at 24 h in leaves (Figure 8B). The expression levels of *ChERF067-2*, *ChERF085-1*, *ChERF097*, *ChERF112-2*, and *ChERF113-1* were shown to be upregulated more than 1.8-fold at 24 h (Figure 9B). For roots, *ChAP2-9*, *ChAP2-10*, *ChAP2-13*, and *ChAP2-22* were upregulated more than 1.4-fold (Figure 7D). *ChERF036-1* was upregulated 2.7-fold at 24 h and *ChERF054-2* was upregulated 6-fold at 6 h (Figure 8D). *ChERF085-1* was upregulated 9-fold at 12 h and *ChERF085-2* was upregulated 8.6-fold at 12 h and 24 h (Figure 9D). In summary, qRT–PCR detection showed that, under 100 μgSe/L and 80,000 μgSe/L selenite treatments, different *ChAP2/ERF* genes in *C. hupingshanensis* leaves and roots exhibited distinct expression patterns.

### 3.7. Prediction and Analysis of ChAP2/ERF Target Genes

**Figure 10:**
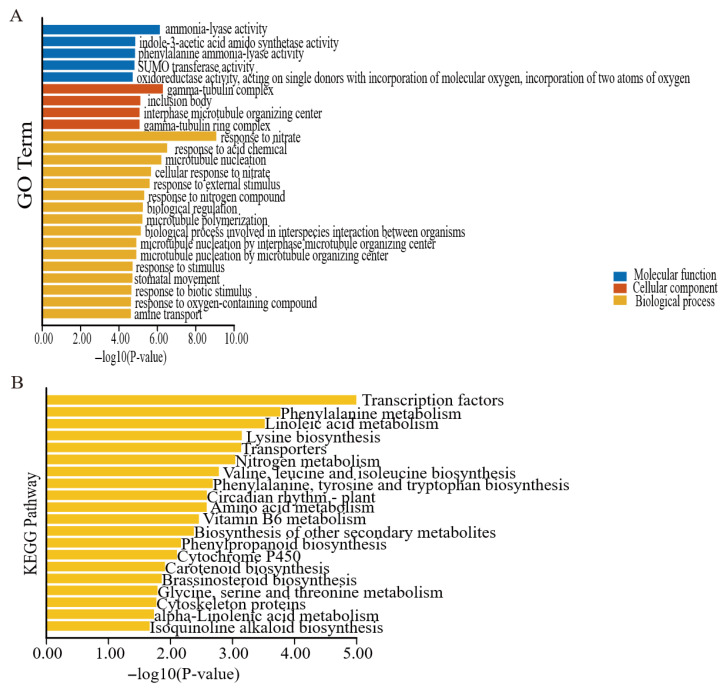
Gene Ontology (GO) terms (**A**) and KEGG pathways (**B**) enriched in *ChAP2/ERF* target genes, illustrating molecular functions, cellular components, biological processes, and metabolic pathway associations.

Given the characteristic of AP2/ERF transcription factors whereby they exert regulatory effects by specifically binding to *cis*-acting elements (such as DRE/CRT, GCC-box, etc.) in the promoter regions of downstream target genes, genes containing either the DRE/CRT element or the GCC-box element in their promoter regions were regarded as potential target genes of *C. hupingshanensis* AP2/ERF transcription factors (*ChAP2/ERF*) in this study. Screening of the *C. hupingshanensis* genome revealed that there were 6678 genes with at least one DRE/CRT element in their promoter regions and 2994 genes with at least one GCC-box element in their promoter regions. By taking the union of these two gene sets, a total of 9672 potential *ChAP2/ERF* target genes were identified, and these genes were used for subsequent Gene Ontology (GO) analysis, and Kyoto Encyclopedia of Genes and Genomes (KEGG) Enrichment analyses of target genes were performed using eggNOG-mapper (http://eggnog-mapper.embl.de/, accessed on 9 December 2024) and visualized using TBtools (https://doi.org/10.1016/j.molp.2020.06.009, accessed on 9 December 2024) (Figure 10B). The GO annotation results of *ChAP2/ERF* target genes showed that they can be divided into three major categories: molecular functions, cellular components, and biological processes. Among them, molecular functions mainly involve SUMO transferase activity, phenylalanine ammonia-lyase activity, etc., indicating that these target genes may participate in metabolic regulation through catalytic reactions, signal molecule synthesis, and other processes. Cellular components are enriched in structures such as interphase microtubule-organizing centers, suggesting that target genes may play a role in processes such as cytoskeleton assembly and organelle function maintenance. Biological processes are mainly involved in responses to nitrogen compounds, etc., reflecting the potential functions of target genes in environmental adaptation, growth and development, and stress responses. The KEGG Enrichment results showed that *ChAP2/ERF* target genes are significantly involved in various metabolic pathways related to amino acid metabolism, other substance metabolism, signal transduction and regulation, functional proteins, and physiological rhythms. In summary, this study identified 9672 potential *ChAP2/ERF* target genes in *C. hupingshanensis* (by screening the genome for genes with DRE/CRT or GCC-box in their promoters) and conducted GO/KEGG analyses, revealing that these targets are involved in molecular functions, cellular components, biological processes, and key metabolic/signaling pathways.

## 4. Discussion

The AP2/ERF transcription factor (TF) family is widely distributed in the plant kingdom and acts as a pivotal regulator of plant growth, development, stress responses, and metabolite biosynthesis [29,30]. Given its functional diversity, genome-wide identification, classification, and functional analysis of this family have been performed in numerous plant species. *C. hupingshanensis*, an endemic *Brassicaceae* selenium (Se) hyperaccumulator, exhibits unique biological traits; it efficiently absorbs, translocates, and safely accumulates Se in high-Se environments while sustaining normal growth, evolving an Se adaptation regulatory network distinct from non-hyperaccumulators [15,31]. However, recent studies on *C. hupingshanensis* Se hyperaccumulation and tolerance mechanisms have not explored the involvement of the AP2/ERF family, a key regulatory factor. Thus, this study conducted genome-wide identification and analysis of the AP2/ERF family in *C. hupingshanensis*. Additionally, quantitative real-time polymerase chain reaction (qRT-PCR) was used to detect the expression patterns of some family members under two different selenium treatments.

Gene family expansion is a crucial evolutionary strategy for plants to cope with specific environmental stresses [3]. In this study, 230 *ChAP2/ERF* genes were identified in *C. hupingshanensis*, and the number was significantly higher than that in non-selenium (Se)-hyperaccumulating plants, such as *A. thaliana*, rice, soybean, maize, grape, and wheat [12,32,33]. Phylogenetic analysis showed that, among the 230 *ChAP2/ERF* genes, the ERF (49.6%) and DREB (30.9%) subfamilies accounted for more than 80% of the total (Section 3.2). This expansion characteristic is consistent with the observations in other cruciferous plants. The high proportion of ERF and DREB subfamilies in *C. hupingshanensis* is not only higher than that in non-Se-hyperaccumulating plants in terms of total gene number but also in subfamily composition. In *A. thaliana* (147 *AP2/ERF* genes) and *Oryza sativa* (163 *AP2/ERF* genes) [4], the combined proportion of ERF and DREB subfamilies is approximately 75% and 65%, respectively—both lower than the 80.5% in *C. hupingshanensis* [7,34]. This selective expansion of ERF and DREB subfamilies further implies their core role in mediating Se stress responses, as these two subfamilies are well known for their functions in rapid abiotic stress signaling and detoxification [35,36], and may be related to the plant’s adaptation to Se stress [37,38]. Meanwhile, subfamilies like AP2 and RAV, though less abundant across species, also have their own significance in plant growth and stress responses [39,40].

From the perspective of chromosomal distribution and gene duplication characteristics, the *ChAP2/ERF* genes are unevenly distributed across chromosomes: chromosomes 8 and 9 contain 25 and 24 genes, respectively. Furthermore, gene duplication events (including segmental duplication and tandem duplication) are more frequent. Specifically, 25 *ChAP2/ERF* genes are distributed on chromosome 8, with a large number of tandem duplication events observed (Figure 3). This finding is consistent with the research results, which points out that stress-related genes in Fagopyrum tataricum enhance adaptability through frequent duplication [12,41]. The expansion of the ERF subfamily and gene duplication events may represent an evolutionary strategy developed by *C. hupingshanensis* to cope with the persistent high selenium stress in its native environment. In addition, 72 pairs of *AP2/ERF* homologous genes were identified between *C. hupingshanensis* and *A. thaliana*, and all 230 *ChAP2/ERF* genes had undergone segmental duplication (Section 3.3), which may contribute to the evolution of selenium adaptation. However, further studies on evolutionary rates and selection pressure are required to confirm this [6,42].

Analysis of 230 *ChAP2/ERF* genes in *C. hupingshanensis* demonstrated that their promoters were significantly enriched with stress-responsive *cis*-acting elements, encompassing those responsive to abscisic acid (ABA), dehydration/cold/salt stress, and light. This finding aligns with the prevalent pattern observed in *AP2/ERF* gene promoters across plants. However, under high selenium stress, the expression activation levels of *ChAP2/ERF* genes harboring these elements were markedly higher than those of their homologous genes in selenium-sensitive species [43]. This observation suggests that the *cis*-acting elements in *C. hupingshanensis* might exhibit higher regulatory efficiency, but this requires verification via promoter activity assays and cross-species comparative analyses. Regarding conserved motifs, motif 1 was highly conserved across all *ChAP2/ERF* proteins. Proteins of the ERF and DREB subfamilies contained a single AP2 domain along with a conserved WLG motif [1,10], and they exhibited the most pronounced expression alterations under high selenium stress. The AP2 subfamily lacked the WLG motif and displayed moderate expression changes. The WLG motif thus may provide a potential basis for subsequent functional exploration [5,44].

In terms of tissue specificity, under low-selenium conditions, the *ChAP2-32* gene was upregulated in roots at 6 h, whereas, under high-selenium conditions, it was upregulated in leaves at 24 h, which is congruent with the selenium accumulation pattern of *C. hupingshanensis* [20]. In contrast, *AP2/ERF* genes in selenium-sensitive plants exhibited consistent downregulation [43]. From a dose-dependent perspective, growth-related ERF genes were more substantially upregulated under low selenium stress, while detoxification-related *AP2/DREB* genes were induced under high selenium stress, which is in line with the bidirectional effects of selenium on plants [45,46]. Concerning subfamily functions, the *ChERF050-1* gene of the DREB subfamily might mitigate selenium-induced reactive oxygen species (ROS) accumulation. Studies have confirmed that genes of the DREB family possess the function of regulating antioxidant enzymes [47,48]. The *ChERF036-1* gene of the ERF subfamily may integrate ethylene signaling with selenium detoxification, as the function of ERF in integrating hormone signals was expounded in the study on ethylene response factors [40,49]. Genes of the RAV subfamily showed moderate expression changes, reflecting their role in balancing growth and stress response. The balancing function of the RAV subfamily in *A. thaliana* root development was referred to in a study on pear RAV transcription factors [9,50]. Furthermore, the functions of each subfamily not only align with the established functions of AP2/ERF subfamilies in other plants but also have undergone adaptive modifications tailored to selenium hyperaccumulation traits [9,39]. In the GO annotation, the molecular function of oxidoreductase activity can maintain cellular redox balance under Se stress. Additionally, the cellular component of the interphase microtubule-organizing center can support the localization and intracellular transport of Se transporters, thereby assisting in Se hyperaccumulation [13,51]. In the KEGG Enrichment analysis, the “cytochrome P450 metabolism” pathway may act as a key detoxification pathway for selenium (Se) speciation transformation. The target genes of this pathway encode cytochrome P450 proteins, which may catalyze the conversion of toxic selenite (SeIV) into low-toxicity selenomethionine (SeMet) and selenocystine (SeCys_2_). This process may provide a potential basis for the safe storage of Se, which is consistent with the low-toxicity Se storage characteristic of *C. hupingshanensis* [20,52]. The target genes of the *ChAP2/ERF* family point to carrier proteins such as those from the SULTR family (responsible for Se uptake in roots) and PCS family (responsible for Se storage in vacuoles). These proteins may help to optimize the inter-tissue/transmembrane transport of Se, thereby avoiding the toxic accumulation of Se in the cytoplasm. This functional role echoes the upregulated expression of genes such as *ChERF085-2* under high-Se conditions [35,53]. The “amino acid metabolism” pathway (phenylalanine and glycine metabolism) may help compensate for the metabolic disorders caused by Se–sulfur competition and scavenge reactive oxygen species (ROS) induced by Se. It may exert these effects by providing precursors for sulfur-containing amino acid synthesis and synergizing with phenylalanine ammonia-lyase to promote the synthesis of antioxidant substances [54,55]. These findings may provide a crucial theoretical basis and clear research directions for subsequent in-depth exploration of the selenium-responsive functions of key genes in this family, as well as the investigation into the evolutionary adaptation strategies underlying plant selenium hyperaccumulation.

## 5. Conclusions

In this study, the AP2/ERF transcription factor family in *C. hupingshanensis* was identified and classified, and the expression patterns of genes in this family under two selenium concentrations (100 μg Se/L and 80,000 μg Se/L) were investigated. This research on the *ChAP2/ERF* family represents the first genome-wide investigation into the AP2/ERF family in the selenium hyperaccumulator *C. hupingshanensis*. The results showed that multiple members of the *ChAP2/ERF* family exhibited tissue-specific, dose-dependent, and temporally dynamic expression responses in leaves and roots under different selenium concentrations, suggesting that these members may be involved in selenium stress responses through pathways such as hormone signal transduction. In conclusion, this study may provide a potential basis for future functional analyses of the *ChAP2/ERF* superfamily. It provides a reference for deciphering the molecular mechanisms underlying selenium stress responses in hyperaccumulating plants.

## Figures and Tables

**Table 1:** The basic physicochemical properties of the *AP2/ERF* family in *C. hupingshanensis*.

Gene ID	Gene Name	DNA Length (bp)	Mature Protein (aa)	pI	MW (kDa)	Grand Average of Hydropathicity (GRAVY)	Subcellular Localization
Chu001362	*ChAP2-1*	972	323	5.27	36.89	−0.887	nucleus
Chu003566	*ChAP2-2*	1575	524	6.4	57.31	−0.685	nucleus
Chu004239	*ChAP2-3*	939	312	6.28	35.53	−1.022	nucleus
Chu004905	*ChAP2-4*	1209	402	5.73	45.90	−0.972	nucleus
Chu007518	*ChAP2-5*	1266	421	8.36	46.55	−0.809	nucleus
Chu007782	*ChAP2-6*	975	324	9.23	36.69	−0.719	nucleus
Chu008869	*ChAP2-7*	1413	470	5.81	51.93	−0.785	nucleus
Chu011886	*ChAP2-8*	1689	562	6.83	61.12	−0.646	nucleus
Chu012136	*ChAP2-9*	1560	519	6.58	56.74	−0.515	chloroplast
Chu012654	*ChAP2-10*	1422	473	5.95	52.63	−0.685	nucleus
Chu012750	*ChAP2-11*	813	270	8.58	30.62	−0.728	nucleus
Chu013398	*ChAP2-12*	1653	550	6.28	60.20	−0.676	nucleus
Chu015708	*ChAP2-13*	942	313	5.43	35.82	−0.993	nucleus
Chu017361	*ChAP2-14*	1299	432	6.6	47.55	−0.728	nucleus
Chu022107	*ChAP2-15*	1647	548	5.83	59.90	−0.732	nucleus
Chu025581	*ChAP2-16*	1050	349	4.87	40.00	−0.667	nucleus
Chu025647	*ChAP2-17*	1050	349	9.39	39.10	−0.566	nucleus
Chu026752	*ChAP2-18*	1635	544	7.14	60.55	−0.694	nucleus
Chu026813	*ChAP2-19*	1269	422	7.75	46.56	−0.788	nucleus
Chu032490	*ChAP2-20*	963	320	7.78	36.56	−0.981	nucleus
Chu033656	*ChAP2-21*	1389	462	6.05	50.98	−0.705	nucleus
Chu033670	*ChAP2-22*	1389	462	6.05	51.01	−0.71	nucleus
Chu034677	*ChAP2-23*	924	307	9.86	34.71	−0.59	nucleus
Chu034915	*ChAP2-24*	1305	434	8.35	48.02	−0.76	nucleus
Chu036333	*ChAP2-25*	1704	567	9.35	63.89	−0.22	extracellular
Chu040760	*ChAP2-26*	1671	556	6.86	60.28	−0.615	nucleus
Chu041002	*ChAP2-27*	1557	518	6.71	56.63	−0.525	chloroplast
Chu041501	*ChAP2-28*	1419	472	5.99	52.57	−0.651	nucleus
Chu041593	*ChAP2-29*	1053	350	6.96	39.43	−0.797	nucleus
Chu042594	*ChAP2-30*	1749	582	6.13	65.10	−0.832	nucleus
Chu046603	*ChAP2-31*	1236	411	5.36	46.22	−0.788	chloroplast
Chu048859	*ChAP2-32*	1872	623	6.86	68.11	−0.738	nucleus
Chu001298	*ChERF001-1*	585	194	6.44	21.08	−0.521	nucleus
Chu015769	*ChERF001-2*	600	199	6.34	21.69	−0.607	nucleus
Chu037160	*ChERF002-1*	762	253	6.75	28.62	−0.861	nucleus
Chu043609	*ChERF002-2*	759	252	6.75	28.62	−0.861	nucleus
Chu037560	*ChERF003-1*	573	190	6.59	21.63	−0.607	mitochondrion
Chu044073	*ChERF003-2*	558	185	7.71	21.01	−0.555	mitochondrion
Chu044144	*ChERF003-3*	459	152	9.58	17.13	−0.632	chloroplast
Chu036394	*ChERF004-1*	570	189	5.89	21.49	−0.636	nucleus
Chu042653	*ChERF004-2*	552	183	9.12	20.74	−0.531	chloroplast
Chu037575	*ChERF005-1*	567	188	9.32	21.21	−0.637	nucleus
Chu003246	*ChERF006*	486	161	9.6	17.96	−0.671	nucleus
Chu039895	*ChERF007-1*	399	132	10.22	14.80	−0.57	peroxisome
Chu039897	*ChERF007-2*	486	161	9.9	18.26	−0.485	peroxisome
Chu009972	*ChERF008-1*	519	172	9.42	18.75	−0.534	chloroplast
Chu032769	*ChERF008-2*	498	165	9.21	17.93	−0.515	chloroplast
Chu017359	*ChERF009*	585	194	7.77	21.23	−0.83	chloroplast
Chu041592	*ChERF010*	546	181	5.87	20.67	−1.103	nucleus
Chu025179	*ChERF011-1*	486	161	9.35	17.99	−0.672	chloroplast
Chu046959	*ChERF011-2*	381	126	10.12	13.93	−0.544	chloroplast
Chu001878	*ChERF012-1*	687	228	6.44	25.58	−0.709	nucleus
Chu015209	*ChERF012-2*	693	230	6.29	25.61	−0.636	nucleus
Chu004423	*ChERF013-1*	690	229	6.18	25.52	−0.498	mitochondrion
Chu032317	*ChERF013-2*	564	187	4.9	20.58	−0.326	mitochondrion
Chu003149	*ChERF014-1*	693	230	7.64	25.19	−0.304	chloroplast
Chu003152	*ChERF014-2*	642	213	6.27	23.16	−0.365	chloroplast
Chu013827	*ChERF014-3*	624	207	5.18	22.75	−0.385	chloroplast
Chu017917	*ChERF015-1*	609	202	5.29	22.52	−0.276	chloroplast
Chu027412	*ChERF015-2*	807	268	8.48	29.87	−0.171	chloroplast
Chu037292	*ChERF016-1*	753	250	4.86	28.28	−0.68	chloroplast
Chu043768	*ChERF016-2*	651	216	4.48	24.54	−0.88	cytosol
Chu001638	*ChERF017-1*	570	189	4.97	21.45	−0.752	cytosol
Chu015431	*ChERF017-2*	573	190	4.89	21.59	−0.764	cytosol
Chu025699	*ChERF018*	576	191	4.61	21.67	−0.517	nucleus
Chu023666	*ChERF019*	423	140	6.03	15.41	−0.549	nucleus
Chu031751	*ChERF020*	426	141	4.94	15.34	−0.579	nucleus
Chu005013	*ChERF021*	564	187	5.22	20.69	−0.543	nucleus
Chu017022	*ChERF023*	561	186	5.49	20.54	−0.458	nucleus
Chu008081	*ChERF024-1*	570	189	4.83	20.40	−0.358	nucleus
Chu034416	*ChERF024-2*	570	189	4.83	20.34	−0.381	chloroplast
Chu001044	*ChERF027-1*	564	187	5.11	20.31	−0.563	nucleus
Chu016014	*ChERF027-2*	546	181	5.07	19.75	−0.598	nucleus
Chu018445	*ChERF029*	663	220	4.73	24.69	−0.422	nucleus
Chu006742	*ChERF032-1*	555	184	5.25	21.05	−0.654	nucleus
Chu030151	*ChERF032-2*	522	173	4.99	19.53	−0.472	nucleus
Chu001043	*ChERF033-1*	618	205	5.25	23.38	−0.632	nucleus
Chu016015	*ChERF033-2*	612	203	5.4	23.23	−0.653	nucleus
Chu007208	*ChERF034-1*	894	297	5	32.18	−0.557	chloroplast
Chu035202	*ChERF034-2*	882	293	5.38	31.59	−0.541	chloroplast
Chu022529	*ChERF036-1*	726	241	4.66	26.66	−0.592	nucleus
Chu049311	*ChERF036-2*	666	221	4.96	24.62	−0.556	nucleus
Chu004460	*ChERF037-1*	681	226	5.05	24.89	−0.581	nucleus
Chu032276	*ChERF037-2*	714	237	4.86	25.97	−0.573	nucleus
Chu034335	*ChERF038*	561	186	5.75	20.75	−0.571	nucleus
Chu019175	*ChERF039-1*	534	177	7.88	19.63	−0.65	nucleus
Chu028753	*ChERF039-2*	534	177	6.97	19.61	−0.655	nucleus
Chu037610	*ChERF040-1*	663	220	5.46	24.05	−0.533	nucleus
Chu044149	*ChERF040-2*	666	221	5.3	24.12	−0.552	nucleus
Chu044192	*ChERF040-3*	666	221	5.3	24.12	−0.552	nucleus
Chu036436	*ChERF041-1*	711	236	5.29	26.09	−0.531	nucleus
Chu042692	*ChERF041-2*	720	239	5.17	26.38	−0.464	nucleus
Chu035916	*ChERF045-1*	990	329	5.12	37.10	−0.885	nucleus
Chu042145	*ChERF045-2*	996	331	4.87	37.43	−0.901	nucleus
Chu007652	*ChERF048-1*	1056	351	4.63	38.96	−0.79	nucleus
Chu034792	*ChERF048-2*	1023	340	4.65	37.74	−0.81	nucleus
Chu032111	*ChERF049*	582	193	5.69	21.32	−0.736	nucleus
Chu037031	*ChERF050-1*	909	302	7.04	33.06	−0.83	nucleus
Chu043367	*ChERF050-2*	900	299	8.55	33.20	−0.816	nucleus
Chu025902	*ChERF051-1*	840	279	5.43	31.67	−0.784	nucleus
Chu046288	*ChERF051-2*	840	279	5.44	31.75	−0.804	nucleus
Chu007663	*ChERF052*	999	332	6.75	36.23	−0.756	nucleus
Chu018190	*ChERF054-1*	849	282	7.1	32.35	−0.994	nucleus
Chu027704	*ChERF054-2*	846	281	5.57	32.33	−0.962	nucleus
Chu032874	*ChERF056*	813	270	5.3	30.47	−0.65	nucleus
Chu015183	*ChERF058*	873	290	5.89	31.87	−0.614	nucleus
Chu004386	*ChERF059-1*	1050	349	6.46	38.19	−0.568	nucleus
Chu032351	*ChERF059-2*	1020	339	6.51	37.20	−0.529	nucleus
Chu017239	*ChERF060-1*	813	270	4.98	30.55	−0.725	nucleus
Chu026680	*ChERF060-2*	795	264	5.25	30.04	−0.763	nucleus
Chu006861	*ChERF061-1*	987	328	8.94	35.99	−0.389	nucleus
Chu030039	*ChERF061-2*	1002	333	7.72	36.56	−0.377	nucleus
Chu019578	*ChERF062*	1017	338	9.36	38.24	−0.513	nucleus
Chu010616	*ChERF063-1*	981	326	4.63	35.95	−0.378	cytosol
Chu039513	*ChERF063-2*	930	309	4.59	33.97	−0.397	nucleus
Chu018603	*ChERF064-1*	999	332	5.17	36.78	−0.527	nucleus
Chu028120	*ChERF064-2*	1029	342	5.25	37.66	−0.475	nucleus
Chu011482	*ChERF065-1*	1038	345	4.59	39.52	−0.723	nucleus
Chu040384	*ChERF065-2*	1059	352	4.61	40.24	−0.759	nucleus
Chu018210	*ChERF066-1*	978	325	4.64	37.19	−0.666	nucleus
Chu027724	*ChERF066-2*	993	330	4.81	37.73	−0.737	nucleus
Chu026223	*ChERF067-1*	933	310	4.8	35.00	−0.725	nucleus
Chu045962	*ChERF067-2*	909	302	4.97	34.39	−0.648	nucleus
Chu007083	*ChERF068-1*	924	307	5.22	34.72	−0.682	nucleus
Chu035331	*ChERF068-2*	924	307	5.21	34.78	−0.7	nucleus
Chu001977	*ChERF069-1*	501	166	9.8	18.23	−0.671	nucleus
Chu015113	*ChERF069-2*	510	169	9.77	18.73	−0.733	mitochondrion
Chu005043	*ChERF070-1*	504	167	9.45	18.63	−0.729	cytosol
Chu031722	*ChERF070-2*	492	163	9.57	18.08	−0.634	chloroplast
Chu006952	*ChERF071-1*	585	194	8.77	21.93	−0.668	cytosol
Chu035447	*ChERF071-2*	525	174	10.23	20.01	−0.498	chloroplast
Chu022480	*ChERF072-1*	726	241	5.25	26.70	−0.568	nucleus
Chu049363	*ChERF072-2*	558	185	10.31	21.22	−0.297	chloroplast
Chu004926	*ChERF073-1*	846	281	5.55	31.66	−0.705	nucleus
Chu031831	*ChERF073-2*	828	277	5.26	31.21	−0.801	nucleus
Chu003796	*ChERF074-1*	1065	354	4.99	39.60	−0.716	nucleus
Chu004086	*ChERF074-2*	1050	349	4.99	39.07	−0.691	nucleus
Chu013182	*ChERF074-3*	999	332	5.31	37.43	−0.747	nucleus
Chu022738	*ChERF075-1*	1173	390	4.97	43.71	−0.878	nucleus
Chu049533	*ChERF075-2*	1158	385	5.01	43.12	−0.864	nucleus
Chu014670	*ChERF076*	498	165	5.55	18.31	−0.388	chloroplast
Chu022641	*ChERF078-1*	663	220	8.99	23.38	−0.499	nucleus
Chu049188	*ChERF078-2*	669	222	9.19	23.53	−0.449	nucleus
Chu003875	*ChERF079*	588	195	9.77	21.30	−0.597	nucleus
Chu038484	*ChERF080-1*	597	198	9.75	21.58	−0.387	nucleus
Chu044580	*ChERF080-2*	588	195	10.04	21.27	−0.313	nucleus
Chu002356	*ChERF081-1*	543	180	9.83	19.80	−0.377	nucleus
Chu014669	*ChERF081-2*	570	189	9.83	20.82	−0.554	nucleus
Chu003526	*ChERF082-1*	693	230	8.86	25.64	−0.748	chloroplast
Chu013438	*ChERF082-2*	705	234	9.05	26.25	−0.651	chloroplast
Chu022161	*ChERF083-1*	768	255	8.83	27.59	−0.691	chloroplast
Chu048935	*ChERF083-2*	765	254	8.93	27.53	−0.728	chloroplast
Chu004193	*ChERF084-1*	792	263	7.75	28.83	−0.476	nucleus
Chu032529	*ChERF084-2*	735	244	9.22	27.16	−0.51	nucleus
Chu036620	*ChERF085-1*	627	208	5.17	23.27	−0.855	nucleus
Chu042903	*ChERF085-2*	636	211	5.2	23.69	−0.838	nucleus
Chu037042	*ChERF086-1*	1044	347	6.03	38.24	−0.62	nucleus
Chu043382	*ChERF086-2*	990	329	6.08	36.15	−0.578	nucleus
Chu002336	*ChERF087-1*	756	251	5.08	28.64	−0.99	nucleus
Chu014738	*ChERF087-2*	756	251	5.07	28.56	1.057	nucleus
Chu002223	*ChERF090-1*	930	309	6.84	34.20	−0.577	nucleus
Chu014844	*ChERF090-2*	930	309	6.31	34.01	−0.588	nucleus
Chu028561	*ChERF091*	471	156	4.94	17.79	−0.484	nucleus
Chu021871	*ChERF092-1*	660	219	5.09	24.82	−0.653	nucleus
Chu048632	*ChERF092-2*	579	192	6.01	22.03	−0.804	nucleus
Chu008600	*ChERF093-1*	762	253	5.42	28.24	−0.719	nucleus
Chu033935	*ChERF093-2*	756	251	6.02	27.93	−0.72	nucleus
Chu000495	*ChERF094-1*	753	250	4.82	28.01	−0.641	nucleus
Chu016638	*ChERF094-2*	699	232	5.29	26.26	−0.684	nucleus
Chu021873	*ChERF095-1*	417	138	5.3	15.66	−0.925	nucleus
Chu048634	*ChERF095-2*	420	139	5.13	15.69	−0.949	nucleus
Chu038373	*ChERF096-1*	390	129	6.42	14.08	−0.961	nucleus
Chu044712	*ChERF096-2*	660	129	6.43	14.16	−0.982	nucleus
Chu016812	*ChERF097*	600	199	9.89	22.84	−0.808	nucleus
Chu021872	*ChERF098-1*	483	160	9.39	18.63	−0.617	nucleus
Chu048633	*ChERF098-2*	435	144	8.85	16.58	−1.102	nucleus
Chu007217	*ChERF099-1*	705	234	5.62	26.23	−0.669	nucleus
Chu035193	*ChERF099-2*	672	223	5.62	25.05	−0.728	nucleus
Chu019108	*ChERF100-1*	792	263	8.98	28.91	−0.523	nucleus
Chu028675	*ChERF100-2*	762	253	9.02	27.87	−0.601	nucleus
Chu038804	*ChERF101-1*	702	233	8.68	25.86	−0.598	nucleus
Chu045021	*ChERF101-2*	741	246	5.88	26.68	−0.473	nucleus
Chu038805	*ChERF102-1*	933	310	5.28	35.04	−0.618	nucleus
Chu045022	*ChERF102-2*	927	308	5.27	34.70	−0.568	nucleus
Chu028676	*ChERF103*	912	303	5.06	34.31	−0.611	nucleus
Chu041142	*ChERF104*	687	228	8.57	25.67	−0.878	nucleus
Chu011284	*ChERF105-1*	654	217	8.93	24.14	−0.659	nucleus
Chu040176	*ChERF105-2*	654	217	8.91	24.35	−0.675	nucleus
Chu032637	*ChERF106-1*	606	201	4.89	22.64	−0.692	nucleus
Chu036108	*ChERF106-2*	606	201	4.89	22.70	−0.674	nucleus
Chu042345	*ChERF106-3*	612	203	4.84	22.82	−0.67	nucleus
Chu003113	*ChERF108-1*	552	183	6.08	20.77	−0.721	nucleus
Chu013905	*ChERF108-2*	831	276	6.24	31.53	−0.603	plasma membrane
Chu013923	*ChERF108-3*	522	183	8.65	20.79	−0.633	nucleus
Chu017615	*ChERF109-1*	783	260	5.19	28.89	−0.695	nucleus
Chu027078	*ChERF109-2*	810	269	5.44	29.75	−0.752	nucleus
Chu011080	*ChERF110-1*	684	227	5.61	24.64	−0.529	nucleus
Chu040012	*ChERF110-2*	612	203	6.21	22.27	−0.567	nucleus
Chu012581	*ChERF111-1*	1176	391	6.81	43.23	−0.915	nucleus
Chu041424	*ChERF111-2*	1194	397	7.89	43.57	−0.84	nucleus
Chu008354	*ChERF112-1*	631	209	7.74	23.34	−0.812	nucleus
Chu034160	*ChERF112-2*	597	198	6.59	22.23	−0.622	nucleus
Chu036565	*ChERF113-1*	648	215	9.22	24.68	−1.175	nucleus
Chu042845	*ChERF113-2*	654	217	6.85	24.54	−1.072	nucleus
Chu012300	*ChERF114-1*	747	248	5.25	27.77	−1.081	nucleus
Chu041160	*ChERF114-2*	720	239	5.19	26.54	−1.139	nucleus
Chu036082	*ChERF115-1*	816	271	7.75	30.40	−0.921	nucleus
Chu042319	*ChERF115-2*	780	259	6.52	29.16	−1.062	nucleus
Chu014898	*ChERF116*	867	288	4.76	31.90	−0.408	nucleus
Chu005439	*ChERF118-1*	996	331	4.74	36.72	−0.507	nucleus
Chu031489	*ChERF118-2*	1002	333	4.62	36.91	−0.555	nucleus
Chu024224	*ChERF119-1*	984	327	5.04	35.97	−0.427	nucleus
Chu026442	*ChERF119-2*	584	327	5.04	36.06	−0.443	nucleus
Chu045481	*ChERF119-3*	1005	334	5.05	37.12	−0.485	nucleus
Chu045483	*ChERF119-4*	1020	339	4.99	37.34	−0.432	nucleus
Chu007757	*ChERF120*	492	163	9.13	18.09	−0.891	nucleus
Chu012767	*ChERF121*	483	160	7.72	17.39	−0.811	nucleus
Chu001099	*ChRAV1*	1020	339	9.19	37.97	−0.666	nucleus
Chu005275	*ChRAV2*	1053	350	9.5	39.44	−0.543	nucleus
Chu024242	*ChRAV3-1*	1026	341	9.14	38.58	−0.681	nucleus
Chu045469	*ChRAV3-2*	1029	342	8.64	38.58	−0.635	nucleus
Chu002169	*ChRAV4-1*	1092	363	9.29	40.44	−0.656	nucleus
Chu014907	*ChRAV4-2*	1095	364	9.1	40.82	−0.673	nucleus
Chu003530	*ChRAV5-1*	1089	362	7.03	41.50	−0.642	nucleus
Chu013433	*ChRAV5-2*	819	272	9.08	31.33	−0.713	nucleus
Chu013434	*ChRAV5-3*	1245	414	8.47	47.80	−0.583	nucleus
Chu003556	*ChRAV6-1*	1020	339	6.57	38.99	−0.738	nucleus
Chu013407	*ChRAV6-2*	804	267	7.69	30.76	−0.596	cytoskeleton
Chu019716	*ChSoloist1*	702	233	9.36	27.49	−1.118	nucleus
Chu029199	*ChSoloist2*	645	214	9.82	25.14	−1.038	chloroplast

## Data Availability

The data that support the findings of this study are available from the corresponding author upon reasonable request.

## Supplementary Materials

The following supporting information can be downloaded at: https://www.mdpi.com/article/10.3390/biology14121686/s1, Table S1: The coding sequences and protein sequences of *ChAP2/ERF* genes; Table S2: The primers of genes involved in *ChAP2/ERF* for qRT-PCR.
